# Lanadelumab in Hereditary Angioedema: Real-World Outcomes and Implications for Access Practices in Europe

**DOI:** 10.3390/jcm15010189

**Published:** 2025-12-26

**Authors:** Dagmara Różyk, Witold Wrona, Barbara Kucharczyk, Agata Tomaszewska, Aleksandra Kucharczyk

**Affiliations:** 1 Law Office Dagmara Różyk Attorney at Law, 00-127 Warsaw, Poland; 2 HealthQuest Sp. z o.o., 01-517 Warsaw, Poland; 3 Medical Faculty I, Medical University of Warsaw, 02-091 Warszawa, Poland; 4 Department of Paediatrics, Nephrology and Allergology, Military Institute of Medicine-National Research Institute, 04-141 Warsaw, Poland; 5 Faculty of Medicine, University of Warsaw, 00-927 Warsaw, Poland; 6 Department of Internal Diseases, Pneumonology, Allergology, Clinical Immunology and Rare Diseases, Military Institute of Medicine-National Research Institute, 04-141 Warsaw, Poland

**Keywords:** hereditary angioedema, lanadelumab, real-world evidence, reimbursement

## Abstract

**Background/Objectives**: Lanadelumab is approved in the EU for long-term HAE prevention in patients aged ≥2 years. While trials show high efficacy, real-world data on reimbursement and outcomes are limited. This study presents real-world clinical results in Poland and compares reimbursement criteria across European countries, assessing how effectiveness influences access restrictions. **Methods**: This retrospective analysis examined patients in the Polish drug program for lanadelumab. It collected demographics, disease features, attack frequency, and rescue medication use before and after at least six months of treatment. Additionally, a review of European reimbursement policies was conducted using health technology assessments, policy documents, and literature. **Results**: The data of 72 patients with HAE with C1 inhibitor deficiency were analyzed. The median follow-up was 20.0 months (IQR 15.0–25.0). The median baseline attack frequency was 15 over 6 months. After 6 months of lanadelumab, attacks dropped to 0 (IQR 0.0–0.0; *p* < 0.001), with 77.8% achieving >90% reduction. Most remained attack-free beyond 6 months; on-demand medication use decreased from 16 to 0 doses (*p* < 0.001). Outcomes persisted beyond 6 months. No demographic or baseline variables predicted response. No discontinuations due to adverse events. Reimbursement criteria across Europe vary, from broad access to restrictions based on attack frequency or treatment response, with differences in care settings. **Conclusions**: Data from Poland confirm lanadelumab nearly eliminates severe HAE attacks in practice, regardless of attack frequency. Some European reimbursement models may exclude patients who could benefit. Using real-world effectiveness evidence in policies could improve access and outcomes for HAE patients.

## 1. Introduction

Hereditary angioedema (HAE) is a rare genetic disorder characterized by recurrent episodes of painful, disabling, and even life-threatening subcutaneous or submucosal edema. Attacks are unpredictable in frequency and severity, imposing significant physical and psychosocial burdens on patients’ daily lives [1]. All patients require access to on-demand therapy, and short-term prophylaxis may be necessary in specific situations (e.g., medical or dental procedures). Long-term prophylaxis (LTP) is a crucial component of HAE management, recommended not only for patients with frequent or severe attacks but also in cases where attacks are unpredictable, associated with high psychosocial burden, or involving potentially life-threatening locations such as the larynx. Until recently, LTP options were limited mainly to tranexamic acid or attenuated androgens, both associated with variable efficacy and safety/tolerability profiles. The introduction of subcutaneous plasma-derived C1-INH and lanadelumab has markedly improved the efficacy and safety of long-term prophylaxis in HAE patients [2,3,4]. Lanadelumab is a fully human monoclonal antibody that inhibits plasma kallikrein and prevents bradykinin-mediated angioedema. Approved in 2018 in the European Union for patients aged ≥12 years and extended in 2023 to children ≥ 2 years [5], lanadelumab was shown to reduce attack frequency by an average of 87.4%, and to enable sustained attack-free periods for many patients [6]. In the HELP extension study, 81.8% of patients were attack-free for ≥6 months on therapy [6], confirming its durable clinical benefit. Reflecting this efficacy, international guidelines position lanadelumab among first-line options for LTP in HAE types I and II [7,8].

However, lanadelumab is a high-cost orphan drug, and its adoption in real-world practice has been shaped by health technology assessments and reimbursement decisions. Many payers have introduced eligibility criteria to target lanadelumab to patients with the most severe disease, aiming to balance cost-effectiveness and budgetary constraints. In Poland, lanadelumab was introduced in 2021 under a national drug program with strict entry criteria requiring ≥12 severe HAE attacks involving the abdomen, pharynx, or larynx, with documented on-demand medication use over 6 months. In 2025, these criteria were modified to ≥6 severe attacks within 6 months, regardless of location, thereby expanding patient access.

Real-world evidence is critical for determining whether lanadelumab’s effectiveness in clinical practice mirrors that observed in clinical trials and for informing future healthcare policy. The study aimed to evaluate real-world clinical outcomes of lanadelumab used for LTP of HAE in patients treated within the national drug program. It also aimed to compare reimbursement criteria across selected European countries and assess whether observed real-world effectiveness aligns with, or challenges, existing access restrictions.

## 2. Materials and Methods

### 2.1. Study Design

This study is a retrospective observational analysis of patients with HAE treated with lanadelumab (Takeda, Tokyo, Japan) in Poland’s nationwide therapeutic program (drug program B.122) for prophylaxis of recurrent angioedema attacks between September 2021 and July 2025. The program enrolled patients aged ≥12 years with HAE type I or II diagnosed based on clinical presentation and confirmed by laboratory tests of C1-INH concentration and activity, C4 and/or C1q (in cases of negative family history in patients > 40 years old) concentrations, according to international guidelines [7]. All patients included in this analysis met the original entry criteria of the program, which required ≥12 severe angioedema attacks within the preceding 6 months, involving the abdomen, pharynx, or larynx, together with documented use of on-demand medication. Although in 2025 the eligibility threshold was formally reduced to ≥6 severe attacks regardless of location, no patients qualifying under these revised criteria had yet been enrolled during the study period. All patients received lanadelumab 300 mg subcutaneously every 2 weeks. Dose interval prolongation (every four weeks) could be considered in patients who did not experience attacks during the first 6 months of treatment. Efficacy was assessed every 6 months based on attack frequency and on-demand medication use, as recorded in program documentation.

### 2.2. Variables

Patient data were collected exclusively from the mandatory monitoring system of the national drug program, with all personal identifiers removed. The following variables were extracted: demographics (age, sex); HAE characteristics (type I or II, family history); clinical history (age at symptom onset, age at diagnosis, prior use of long-term prophylaxis); disease burden (attack frequency, use of on-demand medication); and laboratory findings (C1-INH concentration and activity, C4 concentration, and C1q where indicated by diagnostic criteria).

### 2.3. Outcomes

The primary outcome was the change in the frequency of severe angioedema attacks after the initiation of lanadelumab prophylaxis. A severe attack was defined as an episode involving the abdomen, pharynx, or larynx that required treatment with on-demand medication, i.e., plasma-derived C1-inhibitor or icatibant. The number of severe attacks in the 6 months preceding lanadelumab initiation was compared with the number recorded during the first 6 months of treatment. Secondary outcomes included the reduction in the use of on-demand medication over the same periods and the proportion of patients achieving attack-free status (no severe angioedema attacks during treatment). Additionally, the time course of response was evaluated across successive monitoring visits (every 6 months), reporting the proportion of patients who experienced at least 1 severe attack.

### 2.4. Statistics

Continuous variables were expressed as mean ± standard deviation (SD) or median with interquartile range (IQR), depending on distribution, assessed by the Shapiro–Wilk test. Nominal variables were presented as counts and percentages. For group comparisons, Student’s *t*-test for normally distributed data was used and the Mann–Whitney U or Wilcoxon signed-rank test for non-normal data. We used the Chi-square test to compare proportions. Statistical significance was set at *p* < 0.05 (two-sided).

To explore potential predictors of achieving attack-free status at 6 months of treatment, an exploratory logistic regression analysis was performed. Age and body mass index (BMI) were analyzed as continuous variables. Other covariates (sex, HAE type, use of on-demand medication, baseline attack rate, C1-INH concentration, and C1-INH activity) were analyzed as categorical variables, based on clinically relevant thresholds or data-driven cut-offs.

### 2.5. Review of Reimbursement Criteria in European Countries

Reimbursement policies for lanadelumab were reviewed in Germany, Spain, Italy, France, Belgium, The Netherlands, and the Czech Republic using a targeted search strategy. We retrieved information from official HTA appraisals and payer/government decision documents available on national agency websites, supplemented by peer-reviewed literature (PubMed) and web searches. Searches were performed in July 2025, using predefined keywords, and documents were included if they reported reimbursement status and/or explicit access criteria. For each country, the following variables were extracted: year of reimbursement initiation and funding mechanism; clinical eligibility criteria; patient co-payment or cost-sharing requirements; positioning of lanadelumab within treatment lines; and the healthcare setting of drug provision (ambulatory or home use vs. hospital-only distribution).

## 3. Results

### 3.1. Patient Characteristics

A total of 72 patients met the inclusion criteria, of whom 66 had received lanadelumab for at least 6 months and were evaluable for efficacy. Table 1 presents the baseline characteristics of the patients. A positive family history of HAE was reported in most patients, consistent with the autosomal dominant inheritance pattern. First symptoms appeared in childhood in 95.5% of patients, while only 3 reported onset after 18 years of age. Diagnosis was commonly delayed, with a median interval from symptom onset to confirmed diagnosis of 16.0 years (IQR, 7.8–25.5). The median time from diagnosis to initiation of lanadelumab was 9.0 years (IQR, 5.6–17.5). Approximately one-third of patients had received long-term prophylaxis before starting lanadelumab, most commonly tranexamic acid (*n* = 15) or attenuated androgens (*n* = 14, mainly danazol). All patients had a history of severe angioedema, most frequently abdominal attacks. Laryngeal edema, a life-threatening manifestation, had occurred in 47% of patients at least once, and 31% reported pharyngeal swelling. In the 6 months prior to lanadelumab initiation, patients continued to experience frequent attacks despite access to on-demand treatment, predominantly abdominal.

### 3.2. Efficacy Outcomes

Efficacy was assessed in 66 patients treated with lanadelumab for ≥6 months. Treatment resulted in a marked reduction in severe angioedema attacks. Before initiation, the median attack frequency was 15 per 6 months (IQR, 13.0–20.8). During the first 6 months of therapy, the median number of attacks decreased to 0 (IQR, 0.0–0.0; *p* < 0.001). Attack-free status was achieved in 52 patients (78.8%), while 56 (84.9%) experienced a >90% reduction in severe attack frequency; 4 (6.1%) achieved an 80–90% reduction, and 5 (9.1%) had a <80% reduction.

**Figure 1:**
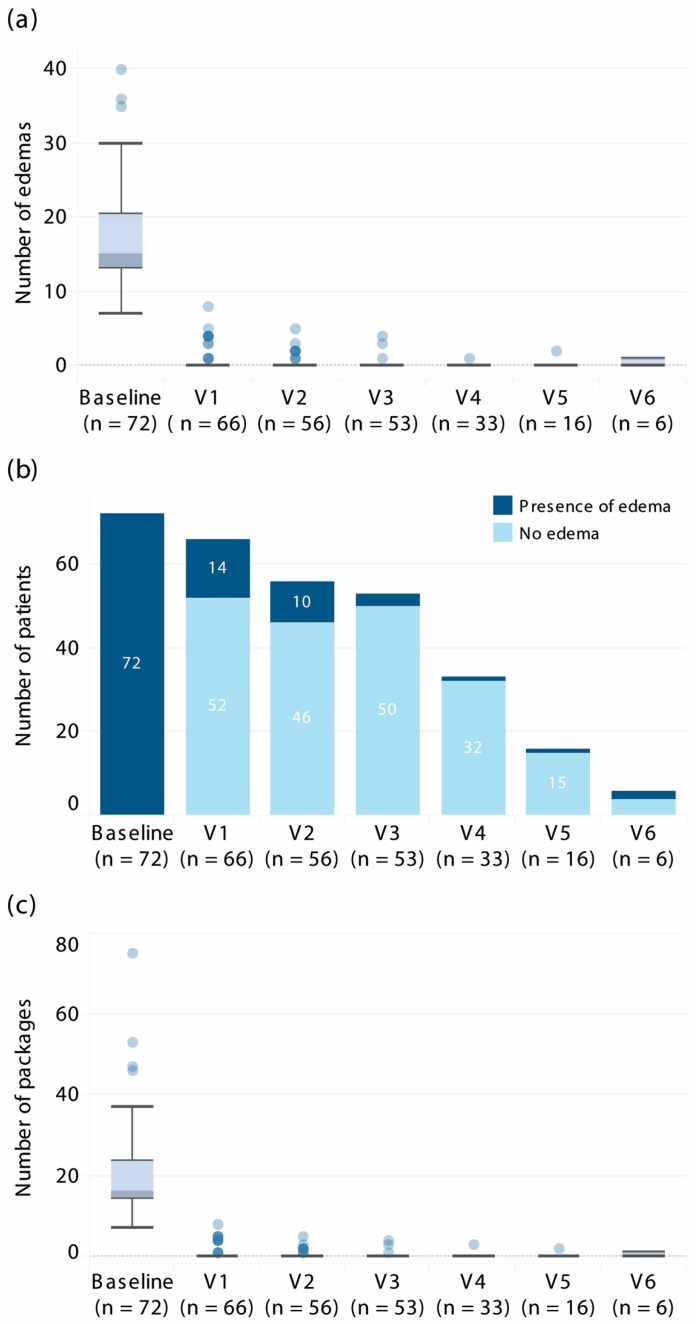
Long-term outcomes of prophylactic use of lanadelumab in Poland. (**a**) Box and whisker plot for a change in the number of abdominal, laryngeal, or pharyngeal attacks from baseline to subsequent follow-up visits. (**b**) Number of patients experiencing any severe HAE attacks at baseline and at follow-up visits. (**c**) Box and whisker plot for a change in the number of packages of on-demand medication used at baseline and during follow-up.

The median follow-up was 20.0 months (IQR, 15.0–25.0). The therapeutic effect was sustained over time (Figure 1). Once response was achieved, most patients remained attack-free at subsequent visits, with the median attack count consistently 0 (IQR, 0.0–0.0) (Figure 1). The proportion of attack-free patients gradually declined at later visits (*p* < 0.001, Chi-square test) (Figure 1).

In parallel, the use of on-demand therapy decreased substantially. Before prophylaxis, patients required frequent on-demand medication (Table 1). After 6 months of treatment with lanadelumab, the median number of on-demand medication uses was 0 (IQR 0.0–0.0; *p* < 0.001), with only 14 patients (19.4%) requiring any on-demand treatment. On-demand use remained negligible beyond the initial 6 months (Figure 1).

One patient discontinued treatment for non-medical reasons (patient decision).

### 3.3. Predictors of Response

Given the uniformly strong efficacy of lanadelumab, no statistically significant predictors were identified that could differentiate patients who achieved attack-free status (100% reduction in severe attacks) from those with partial responses. Multivariate logistic regression did not reveal any baseline demographic or clinical variables significantly associated with reduced likelihood of response (*p* > 0.05 for all covariates examined, Table 2). Lanadelumab was consistently effective across all analyzed subgroups, including age, sex, BMI, HAE type, baseline attack frequency, and prior use of long-term prophylaxis.

### 3.4. Reimbursement Criteria for Lanadelumab in Selected European Countries

Table 3 lists key reimbursement and access parameters for lanadelumab across several European countries. All listed countries have national health insurance systems that fund lanadelumab for HAE LTP, generally with full coverage and no patient co-payments, aside from minimal prescription fees or deductibles, in line with rare disease policies.

#### 3.4.1. Availability and Reimbursement Process

In Germany, newly approved drugs are automatically reimbursed; lanadelumab became available in 2019 [10] and was formally assessed by the Federal Joint Committee (G-BA) in 2021 [11]. Although the G-BA concluded that lanadelumab offers no additional benefit compared with the appropriate comparator therapy (subcutaneous plasma-derived C1-INH, considered first-line prophylaxis), the drug continues to be reimbursed owing to its orphan drug status. In the Czech Republic, the reimbursement process was the most prolonged, reflecting both high therapy costs and limited resources for other LTP options. Until 2023, lanadelumab access had been restricted to case-by-case financing [18]; regular reimbursement began in 2024.

#### 3.4.2. Eligibility Criteria

Requirements vary substantially between countries. Liberal policies are observed in Germany, The Netherlands, and Spain, where no strict numeric thresholds are imposed and specialist judgment predominates [12,16]. In contrast, Poland, Belgium, France, and the Czech Republic apply restrictive entry criteria, typically requiring documentation of attack frequency [9,15,17,18]. Poland initially set the strictest threshold (≥12 attacks in 6 months), which was reduced in 2025 to ≥6 attacks. While the EMA label allows use in children aged ≥2 years [5], reimbursement in many countries remains limited to patients aged ≥12 years (Table 3).

#### 3.4.3. Positioning Within Treatment Lines

In most countries, lanadelumab is available as a first-line prophylactic option. However, in France and Italy, reimbursement criteria explicitly restrict its use to second-line therapy. In these settings, patients must have failed or not tolerate older prophylactic therapies (tranexamic acid, attenuated androgens), and in practice, lanadelumab can be prescribed only after subcutaneous plasma-derived C1-INH has been considered or used [13,14] (Table 3).

#### 3.4.4. Distribution Setting

In Germany and the Netherlands, lanadelumab is dispensed via outpatient pharmacies, whereas its distribution is restricted to hospital-based programs in Poland, Belgium, Italy, Spain, and France.

## 4. Discussion

Real-world data from Poland strongly reinforce the clinical trial evidence that lanadelumab is a highly effective LTP for HAE. In this analysis, one of the largest real-world HAE cohorts reported from a single country, lanadelumab essentially abolished angioedema attacks in most patients. The median attack frequency fell to zero, and more than three-quarters of patients achieved >90% reduction in severe attacks. These outcomes are comparable to, or even exceed, those reported in clinical trials and open-label extension studies. In the HELP study’s open-label extension, the mean reduction in attacks was 87.4%, and 81.8% of patients remained attack-free for ≥6 months [6]. In the EMPOWER study, patients who continued therapy for 3 years had a mean of 0.2 attacks per month (95% CI, 0.1–0.3) [19]. In the INTEGRATED cohort study, which was conducted in Germany, France, Austria, and Greece, the attack-free rate rose from 0% to 54.4% after 12 months of treatment [20].

A key distinction between our cohort and earlier reports is the selection of more severely affected patients in the Polish national program. The program’s initial criteria required a median of multiple severe attacks within the preceding 6 months, reflecting highly active disease. In contrast, in the HELP, EMPOWER, and INTEGRATED studies, most attacks were mild or moderate [6,19], or the overall burden of severe attacks was low (e.g., a mean of 2.8 attacks per year [20]). The near-complete suppression of severe attacks observed in our study may therefore reflect both the efficacy of lanadelumab and the very high baseline disease activity.

Findings from the French compassionate early-access program support this interpretation: patients had a median of 13.5 attacks (range, 1–99) with a high proportion of severe episodes, and 66% were attack-free after 6 months of lanadelumab [21]—a result closely aligned with our observations. In Poland, program eligibility has recently been revised to include patients with less frequent attacks; however, these individuals were not analyzed here because they had not yet reached 6 months of follow-up at the time of data collection. Importantly, our analysis revealed no predictors of poor response. Even patients with relatively lower baseline attack rates achieved remission, supporting the decision to broaden access so that a wider group of HAE patients can benefit from effective prophylaxis.

In some European countries, such as Poland and Belgium, reimbursement authorities initially imposed very strict eligibility criteria for lanadelumab. This cautious approach reflected the limited long-term real-world effectiveness and cost-effectiveness data available at the time of approval. For example, in 2020, the Polish HTA Agency issued a negative recommendation, citing concerns about budget impact and uncertainty in extrapolating trial data to broad real-life populations [21]. Reimbursement was eventually granted in 2021 following negotiations and recognition of HAE as an ultra-rare disease, but with stringent entry conditions and a time-limited agreement. Over time, as robust real-world evidence has accumulated, these restrictions have been progressively relaxed. In Poland, the initial requirement of ≥12 attacks in 6 months was reduced to ≥6 attacks starting in January 2025, enabling wider patient access [22]. In France, restrictions on second-line use reflected uncertainty regarding benefits in LTP-naive patients [14]. Overall, payer decisions across Europe—including Poland, France, Germany, and Spain—have increasingly been shaped by emerging real-world data [10,22,23,24].

Paradoxically, the high effectiveness of lanadelumab does not automatically translate into broad patient access. Even though HAE is a rare disease with an estimated prevalence of 1 per 10,000 to 1 per 150,000 persons [25], the overall demand for this costly therapy can be substantial, justified by the proven attack-free rates and high levels of patient satisfaction [26,27]. Payers defining eligibility criteria for therapies financed from public sources aim primarily to contain budget impact, which is strongly influenced by the number of patients treated. In Belgium, for example, with an estimated 230 patients (≈1 per 50,000 population), the reimbursement criteria restricted treatment to only 20 individuals in 2022 [28].

However, while prioritizing patients with the greatest medical need may appear reasonable, it raises important questions of fairness and medical ethics. Should patients truly be required to endure a certain number of attacks before being granted access to a treatment that could almost completely prevent them? This dilemma is especially relevant in the context of HAE’s chronic and unpredictable nature. Denying or delaying prophylaxis until patients “qualify” by suffering enough attacks can be regarded as inequitable.

The rollout of lanadelumab across European health systems illustrates how contextual factors, such as financing arrangements, healthcare infrastructure, and stakeholder priorities, shape the adoption of evidence-based interventions. In countries such as Germany and The Netherlands, the context is characterized by robust healthcare funding and strong reliance on specialist physicians to manage rare-disease therapies. Consequently, lanadelumab has been introduced with minimal administrative barriers, leaving clinicians to identify appropriate patients. This approach maximizes access and is likely to improve outcomes at the population level. The Dutch authorities explicitly stated that no strict eligibility thresholds were required, as the drug has no equivalent alternative and should be available without restrictions [29]. However, this policy has not resulted in large numbers of treated patients. On the basis of 2022 prevalence estimates of ~1000 individuals with HAE in The Netherlands, approximately 20 patients were reported as receiving lanadelumab, with several more in the qualification process; this figure was confirmed by Dutch experts in 2025 (personal communication), indicating that uptake remains limited to date [28]. The expected steady-state treatment population is projected to reach ~80, including those anticipated to switch from older LTP agents [28]. This suggests that clinical guidelines and specialist oversight can serve as effective gatekeepers, potentially achieving a balance between access and cost control through professional self-regulation.

In countries such as Italy and France, reimbursement policies stipulate that patients must first fail treatment with attenuated androgens and C1-INH before lanadelumab can be prescribed. In France, the second-line restriction reflects the absence of direct head-to-head comparisons with other prophylactic agents [14]. However, such line-of-therapy rules may conflict with the principle of tailoring long-term prophylaxis to individual disease burden and patient preference [7]. Our data suggest that lanadelumab-based LTP can reduce the drawbacks associated with prolonged androgen exposure [29] and markedly decrease the need for on-demand management of attacks.

In the Czech Republic, a risk-sharing agreement enabled the transition from compassionate use to a sustainable reimbursement model. This illustrates how financial mechanisms can mitigate payer risk. As more real-world outcome data such as ours become available, payers may gain greater confidence that they are funding a therapy with transformative benefits. This, in turn, could support further relaxation of eligibility criteria or the renewal of reimbursement agreements in the coming years.

Our clinical findings have several limitations. Because entry into the Polish drug program required a high baseline attack frequency, our cohort reflects only patients with severe HAE. This likely magnified the apparent treatment benefit, as patients with the greatest disease burden have the most to gain. Therefore, the effect size observed here may not be directly extrapolated to patients with milder disease, in whom the relative reduction per attack avoided would be smaller.

The retrospective, observational design of this study precludes definitive causal inference and limits control over unmeasured variables. Information on country-specific reimbursement policies was derived from publicly available documents and published literature. Finally, we did not conduct an economic evaluation, even though such analyses are often a major factor in reimbursement decisions for high-cost orphan drugs.

Despite limitations, integrating real-world clinical data from a national cohort with policy comparisons provides a unique perspective on both effectiveness and access. This combined approach allows assessment of whether reimbursement restrictions are aligned with patient outcomes. Nevertheless, prospective, multinational studies, including health economic analyses, are needed to bridge the gap between policy and practice.

## 5. Conclusions

The Polish real-world data demonstrate that lanadelumab can virtually eliminate HAE attacks in patients with severe disease, confirming its profound clinical effectiveness. These findings support broader use of lanadelumab prophylaxis, as even patients with moderately frequent attacks are likely to derive substantial benefit. Our comparative analysis of European reimbursement policies shows that many countries adopted a cautious initial approach, largely driven by cost concerns, but that these restrictions are gradually relaxed as real-world evidence accumulates. Shaping access policies requires balancing efficacy, cost, and equity. The high remission rates achieved with lanadelumab directly challenge restrictive reimbursement criteria. The paradox of limiting access to a highly effective therapy because of budget impact must be addressed through innovative funding mechanisms and ongoing policy adjustments. Encouragingly, many countries, including Poland, are already easing eligibility thresholds, and others are implementing outcome-based monitoring. International collaboration and systematic data sharing will be essential to guide best practices for the adoption of life-changing therapies, such as lanadelumab.

## Figures and Tables

**Table 1:** Patient characteristics.

Characteristics	Value
Mean age (±SD), years	38.0 (13.6)
Female sex, *n* (%)	55 (76)
Median BMI (IQR), kg/m^2^	24.8 (22.8–28.4)
HAE type, *n* (%)	
I	67 (93)
II	5 (7)
Median C1-inhibitor level (IQR), g/L	0.05 (0.04–0.07)
Mean C1-inhibitor activity (±SD), %	16.3 (8.1)
Median C4 level (IQR), g/L	0.04 (0.03–0.06)
First-degree relatives diagnosed with HAE, *n* (%)	59 (82)
Median number of attacks six months before treatment (IQR)	
Overall	15.0 (13.0–20.8)
Abdominal attacks	13.0 (10.3–17.0)
Pharyngeal attacks	0.0 (0.0–1.0)
Laryngeal attacks	0.0 (0.0–2.0)
Median age at disease onset (IQR), years	7.5 (5.0–12.0)
Mean age at diagnosis (±SD), years	26.0 (13.7)
Long-term prophylaxis use, *n* (%)	26 (36)
Median number of standard doses of on-demand treatment used in the 6 months before treatment (IQR)	
Overall	16.0 (14.0–23.8)
pdC1-INH 1500 IU	8.0 (2.0–17.0)
Icatibant	8.0 (0.0–13.0)

C1-INH, C1-inhibitor; IQR, interquartile range; HAE, hereditary angioedema; IU, international units; pdC1-INH, plasma-derived C1-inhibitor; SD, standard deviation.

**Table 2:** Multivariate logistic regression analysis of factors predicting a complete reduction (100%) in the frequency of angioedema attacks at six months of treatment.

Variable	B Coefficient	Standard Error	*p*-Value
Age	−0.008	0.760	0.992
Sex	1.754	1.413	0.214
Age of symptom onset	−0.083	1.437	0.954
Time from symptom onset to diagnosis	−0.121	1.445	0.933
Time from symptom onset to treatment	−0.106	1.445	0.942
Height	−0.211	0.530	0.691
Body mass	0.341	0.668	0.610
Body mass index	−0.981	1.880	0.602
C1-INH concentration	0.046	6.948	0.995
C1-INH activity	0.050	0.047	0.285
Baseline frequency of angioedema attacks	0.004	0.114	0.970
Number of treatment lines	0.088	0.092	0.337
Age at treatment with lanadelumab	0.131	1.550	0.933
HAE type	0.208	1.965	0.916

C1-INH, C1-inhibitor; HAE, hereditary angioedema.

**Table 3:** Reimbursement criteria for lanadelumab prophylaxis in hereditary angioedema in selected European countries.

Country	Reimbursement Criteria
**Poland**	Reimbursed since: September 2021.
Eligibility	Patients ≥12 years of age with ≥12 severe abdominal/laryngeal/pharyngeal HAE attacks in 6 months with documented on-demand medication use (2021–2024); threshold relaxed to ≥6 attacks/6 months from 2025 [9].
Patient cost	Free of charge.
**Germany**	Reimbursed since: 2019 [10].
Eligibility	Per EMA indication. In practice, used for patients with frequent or severe attacks; no formal attack-count threshold; no prior prophylaxis required [11].
Patient cost	Statutory co-payment of 10% of the retail price (min €5, max €10 per pack); annual cap 2% of household gross income (1% for chronically ill). Exempt for patients ≤ 18 years.
**Spain**	Reimbursed since: March 2021.
Eligibility	Per EMA indication [12]. Initiated in expert centers, typically for frequent (e.g., ≥1 attack/month) or severe attacks in line with WAO/EAACI guidelines [7]. No prior prophylaxis failure required.
Patient cost	Free of charge.
**Italy**	Reimbursed since: February 2021.
Eligibility	Patients ≥12 years with intolerance or contraindications for danazol-based LTP; requires an AIFA therapeutic plan issued by a specialist in an authorized center; plan valid for up to 12 months with reassessment thereafter. No formal numeric attack-frequency threshold [13].
Patient cost	Free of charge.
**France**	Reimbursed since: 2020.
Eligibility	Patients ≥2 years who failed, did not tolerate, or had contraindications to first-line LTP (attenuated androgens or pdC1-INH); reserved for severe or life-threatening disease. No formal attack-count threshold [14].
Patient cost	Free of charge.
**Belgium**	Reimbursed since: July 2022.
Eligibility	Patients ≥12 years with HAE type I/II who have: (a) >1 severe angioedema attack/month (>5 months/year affected), or (b) ≥1 life-threatening upper-airway swelling, or (c) inadequate control with repeated on-demand therapy. Initial approval is for 3 months; continuation requires ≥50% attack reduction at 3 months. Subsequent authorizations in 12-month cycles with annual specialist re-application and insurer review [15].
Patient cost	Free of charge.
**The Netherlands**	Reimbursed since: 2023.
Eligibility	Covered under basic insurance per EU label; initiation at HAE specialist’s discretion, guided by consensus (prophylaxis typically if ≥1 severe attack/month or significant QoL impact). No step-through required [16].
Patient cost	Covered under basic insurance; patients pay up to the statutory annual deductible (eigen risico, €385 in 2025). Beyond this amount, treatment is fully reimbursed.
**Czech Republic**	Reimbursed since: Regular reimbursement from 2024; previously case-by-case [17].
Eligibility	Czech expert guidance recommends first-line LTP (lanadelumab or pdC1-INH) for (a) ≥18 attacks/year or (b) ≥1 life-threatening attack in the last year, or (c) extremely severe disease (e.g., frequent multifocal attacks, multiple acute doses), or (d) insufficient effect of or contraindication to other prophylaxis [18]. Final official criteria pending publication.
Patient cost	Free of charge.

AIFA, Agenzia Italiana del Farmaco (Italian Medicines Agency); EMA, European Medicines Agency; EU, European Union; HAE, hereditary angioedema; LTP, long-term prophylaxis; pdC1-INH, plasma-derived C1-inhibitor; QoL, quality of life; WAO/EAACI, World Allergy Organization/European Academy of Allergy and Clinical Immunology.

## Data Availability

The datasets analyzed during the current study were obtained from the System Monitorowania Programów Terapeutycznych (SMPT) registry, which is accessible only to the Coordinating Team and the National Health Fund (NFZ). Due to confidentiality and regulatory restrictions, the data are not publicly available but may be accessible from the corresponding author upon reasonable request and with the NFZ’s permission.

## Abbreviations

The following abbreviations are used in this manuscript:

C1-INH	C1 esterase inhibitor
CI	Confidence interval
HAE	Hereditary angioedema
HELP	Hereditary Angioedema Long-term Prophylaxis study
IQR	Interquartile range
LTP	Long-term prophylaxis
SD	Standard deviation
